# Bioproduction of a Therapeutic Vaccine Against Human Papillomavirus in Tomato Hairy Root Cultures

**DOI:** 10.3389/fpls.2019.00452

**Published:** 2019-04-11

**Authors:** Silvia Massa, Francesca Paolini, Carmela Marino, Rosella Franconi, Aldo Venuti

**Affiliations:** ^1^ Biotechnology Laboratory, Biotechnology and Agroindustry Division, Department of Sustainability, ENEA (Italian National Agency for New Technologies, Energy and Sustainable Economic Development), Rome, Italy; ^2^ Virology Laboratory, HPV-UNIT, Department of Research, Advanced Diagnostic and Technological Innovation (RIDAIT), Translational Research Functional Departmental Area, IRCSS Regina Elena National Cancer Institute, Rome, Italy; ^3^ Biomedical Technologies Laboratory, Health Technologies Division, Department of Sustainability, ENEA, Rome, Italy

**Keywords:** plant molecular farming, hairy root cultures, plant-produced antigens, HPV – human papillomavirus, cancer, therapeutic vaccines, heterologous prime – boost

## Abstract

Human papillomavirus (HPV) tumor disease is a critical public health problem worldwide, especially in the developing countries. The recognized pathogenic function of E5, E6, and E7 oncoproteins offers the opportunity to devise therapeutic vaccines based on engineered recombinant proteins. The potential of plants to manufacture engineered compounds for pharmaceutical purposes, from small to complex protein molecules, allows the expression of HPV antigens and, possibly, the regulation of immune functions to develop very specific therapies as a reinforcement to available nonspecific therapies and preventive vaccination also in developed countries. Among plant-based expression formats, hairy root cultures are a robust platform combining the benefits of eukaryotic plant-based bioreactors, with those typical of cell cultures. In this work, to devise an experimental therapeutic vaccine against HPV, hairy root cultures were used to express a harmless form of the HPV type 16 E7 protein (E7*) fused to SAPKQ, a noncytotoxic form of the saporin protein from *Saponaria officinalis,* that we had shown to improve E7-specific cell-mediated responses as a fusion E7*-SAPKQ DNA vaccine. Hairy root clones expressing the E7*-SAPKQ candidate vaccine were obtained upon infection of leaf explants of *Solanum lycopersicum* using a recombinant plant expression vector. Yield was approximately 35.5 μg/g of fresh weight. Mouse immunization with vaccine-containing crude extracts was performed together with immunological and biological tests to investigate immune responses and anticancer activity, respectively. Animals were primed with either E7*-SAPKQ DNA-based vaccine or E7*-SAPKQ root extract-based vaccine and boosted with the same (homologous schedule) or with the other vaccine preparation (heterologous schedule) in the context of TC-1 experimental mouse model of HPV-associated tumor. All the formulations exhibited an immunological response associated to anticancer activity. In particular, DNA as prime and hairy root extract as boost demonstrated the highest efficacy. This work, based on the development of low-cost technologies, highlights the suitability of hairy root cultures as possible biofactories of therapeutic HPV vaccines and underlines the importance of the synergic combination of treatment modalities for future developments in this field.

## Introduction

Over the past four decades, a wealth of literature has demonstrated the production of exogenous proteins in plants for health applications. Indeed, plant-based expression systems have great potential to produce different types of biologics at reasonable costs and with reduced risks of contamination by threatening pathogens. This approach is especially advantageous in the field of prevention and treatment of infections and cancer (Rybicki, 2014; Streatfield et al., 2015; Loh et al., 2017).

Transient expression of target proteins achieved by plant viruses or by agroinfiltration often allows higher protein yield with respect to stable transformation due to the absence of chromosomal integration (Komarova et al., 2010) and represents also a means for evaluation of expression before starting the generation of transgenic plant-based expression platforms. Nevertheless, expression of therapeutic proteins using *in vitro* plant systems under contained conditions represents a profitable manufacturing approach in terms of uniform cultivation conditions, product quality, and downstream purification process (Rischer et al., 2013; Santos et al., 2016; Massa et al., 2018). Together with cell suspensions, organ cultures such as hairy root cultures (HRCs) offer advantages including containment, established cultivation conditions in hormone-free media, product homogeneity (Franconi et al., 2010; Schillberg et al., 2013). Hairy roots are particularly attractive for the industrial-scale production of secondary metabolites (Miralpeix et al., 2013), but are also considered for the expression of pharmaceutical proteins, due to better performances over plant cell suspension cultures in terms of genetic and biochemical stability, reduced presence or absence of toxic compounds, such as alkaloids, with respect to leaves. Among plants used for generating hairy roots, crop plants such as tomato and potato were also used. Indeed, hairy roots of many different plant species have been utilized to produce various both secondary metabolites and recombinant proteins of pharmaceutical value at varying yields, such as enzymes (Woods et al., 2008), vaccines and hormones (Woffenden et al., 2008; Skarjinskaia et al., 2013), antibodies in different formats (Wongsamuth and Doran, 1997; Lonoce et al., 2016, 2018). Production of enzymes for replacement therapy of rare diseases was also reported (Rodriguez-Hernandez et al., submitted; Naphatsamon et al., 2018). Recombinant proteins produced in engineered hairy root cultures can be also secreted in the culture medium simplifying downstream purification processes (Guillon et al., 2006; Häkkinen et al., 2014).

Cervical cancer and cervical intraepithelial neoplasia (CIN) are known consequences of human papillomavirus (HPV) infection. Cervical cancer is the fourth most common cancer in female population, with about 569,847 new cases per year (of which 88% in developing countries) and over 311,365 deaths (GLOBOCAN 2018, https://gco.iarc.fr/today). HPV is also the agent behind the development of other tumors and of oropharyngeal carcinogenesis, now in significant rise, and has a causal role in 13% of all female cancers (i.e., 5% of all cancers). Expression of viral oncogenes such as E6 and E7, and, as it was more recently demonstrated, E5, leads to correlated malignant disease.

Although HPV infection is preventable through very efficient recombinant vaccines developed against variously incident oncogenic genotypes in yeast and insect cells, and despite cervical cytology and DNA testing, HPV-related preinvasive and invasive diseases remain critical public health problems. Furthermore, currently available treatments against HPV-related disease are only moderately successful, with radiotherapy, chemotherapy, and surgery very poorly efficient against high-grade lesions (Vici et al., 2014; Cordeiro et al., 2018). This highlights the need for specific treatment strategies. Among the most promising, there are therapeutic vaccines and novel therapeutics that may target the ability of HPV to influence host immune tolerance. If available, these tools may also imply milder side effects than conventional approaches such as radiotherapy and/or chemotherapy.

Immunological therapeutic approaches against HPV have been investigated in the last decades, facilitated by the availability of E5, E6, and E7 tumor-associated antigens, optimal targets for cancer immunotherapy. First examples of experimental HPV therapeutic vaccines were able to block tumor growth in animal models, with some being able to evoke specific cell-mediated immune responses (Cordeiro et al., 2018). However, poor presentation of viral antigens that are expressed at low levels and poor trafficking of effector T-cell populations to non-inflamed mucosal/skin sites were common limitations. The use of adjuvants was, indeed, demonstrated to be crucial for therapeutic efficacy (Gerard et al., 2001).

Studies have focused on enhanced E6 and E7 HPV peptide-fusions in combination with bacterial toxins and/or adjuvants. Nevertheless, these approaches showed negligible correlation to good clinical outcomes and tumor regression (Skeate et al., 2016). An advantage of whole protein-based vaccines compared to peptides is that they theoretically cover all available cytotoxic T lymphocytes (CTLs) and T-helper epitopes. Therefore, the use of whole HPV E6 and E7 proteins or fusion proteins as the antigenic source has been widely employed in preclinical therapeutic vaccines tested in animal models and advanced into phase II and III clinical trials (Vici et al., 2016; Barros et al., 2018; Roden and Stern, 2018).

Among protein-based formulations, production of candidate HPV therapeutic/prophylactic vaccines using plant-derived expression platforms was also proven. Different plant-based expression systems were considered, from whole plant approaches for transient expression to stably transformed green microalgae, using single HPV antigens or fusion to peptides to improve accumulation yield, to intracellular targeting strategies. In many cases, evidence of immunogenicity and efficacy in animal models were reported (reviewed in Chabeda et al., 2018). In our previous experience, crude plant extracts containing HPV16 E7 antigen were shown to provide protection against challenge with E7-expressing TC-1 cells (Franconi et al., 2002, 2006). These responses were improved when E7 was produced in plants as fusion to a bacterial carrier (LicKM, *Clostridium thermocellum* beta-glucanase) (Massa et al., 2007; Venuti et al., 2009).

Besides protein-based formulations, genetic vaccination is also a promising immunotherapeutic tool due to amenability to engineer sequences (e.g., addition of sequences of immunological value), stability and ease of manufacturing, cost-effectiveness, safety, and general tolerability (Gurunathan et al., 2000). Genetic immunization is able to induce adaptive cell-mediated immune responses, including activation of CD4^+^ helper T cells and CD8^+^ cytotoxic T cells, crucial to the resolution of cancer (Stevenson et al., 2004). Indeed, the most remarkable result in the field of therapeutic HPV vaccines is the clinical activity showed by a phase IIb randomized trial performed with a DNA-based vaccine (VGX-3100) in either HPV16- or HPV18-positive CIN2/3 patients (Trimble et al., 2015). In this trial, DNA vaccine delivery included the use of intramuscular injection coupled with electroporation (i.e., administration of short electrical pulses at the site of the DNA vaccine injection to increase plasmid uptake and correlated immune response) and showed, for the first time, significant regression of CIN2 lesions.

In our search for innovative immune-stimulatory tools for the rational design of therapeutic DNA-based vaccines against HPV (Massa et al., 2008), we focused on the sequence encoding the saporin protein (SAP) from *Saponaria officinalis*, a member of the “Ribosome-Inactivating Proteins” (RIPs) family (Hartley and Lord, 2004; Stirpe, 2004; Zarovni et al., 2009). We demonstrated that the combination of a mutagenized SAP sequence (SAPKQ) with an attenuated, synthetic HPV16 E7 gene (E7GGG, thereafter indicated as E7*), in the context of DNA-based vaccination, determined a modulation of E7-specific humoral and cell-mediated immune responses affecting the growth of E7-expressing tumors (Massa et al., 2011).

Heterologous DNA prime-protein boost regimen (i.e., administration of an immunogen as a DNA-based preparation in the first dose, followed by the same immunogen as a protein-based preparation in the booster dose) is emerging as a tool for envisaging new therapeutic options in HPV-associated infection and cancer (Peng et al., 2016).

In the present study, hairy root cultures derived from tomato (*Solanum lycopersicum*) were used to stably express E7*-SAPKQ in order to devise a protein-based experimental HPV therapeutic vaccine. HRC turned out to be a better platform to express E7*-SAPKQ than *E. coli*, due to its ability to accumulate the recombinant vaccine in the soluble fraction of root extracts. The administration of the DNA-based E7*-SAPKQ vaccine (prime dose) followed by E7*-SAPKQ protein-containing hairy root extract (boost dose) was considered, together with homologous prime-boost regimens. All the formulations exhibited an immunological response associated to anticancer activity. In particular, DNA as prime and hairy root extract as boost demonstrated the highest efficacy.

This work, based on the development of low-cost technologies (i.e., DNA-based vaccination and plant-based expression systems), highlights the suitability of hairy root cultures as possible biofactories of therapeutic HPV vaccines and underlines the importance of the synergic combination of treatment modalities for future developments in this field.

## Materials and Methods

### Cells

*Agrobacterium tumefaciens* C58C1 strain was used for plant-based transient expression experiments. *Agrobacterium rhizogenes* A4 (*Rhizobium rhizogenes* ATCC 43057; American Type Culture Collection, Manassas, VA, USA) was used to generate hairy root clones. Bacteria were grown in YEB medium (5 g/l beef extract, 1 g/l yeast extract, 5 g/l peptone, 5 g/l sucrose, 2 mM MgSO_4_) at 28°C with shaking at 220 rpm. When necessary, kanamycin (50 μg/ml) was added to the culture medium.

E7-expressing TC-1 tumor cells were kindly gifted by T.C. Wu (Johns Hopkins Medical Institutions, Baltimore, MD) and were cultivated in RPMI (Invitrogen, Paisley, UK) containing 400 μg/ml G418 and 10% fetal calf serum.

### Animals

Six- to eight-week-old female C57BL/6 mice were supplied by Charles River Laboratories. Animal handling and sacrifice were performed under specific pathogen-free conditions at the Animal House of the Regina Elena National Cancer Institute. All experimental procedures were approved by the Government Committee of National Minister of Health (85/2016-PR) and were carried out in accordance with EU Directive 2010/63/EU for animal experiments.

### Plant Material, Genes, and Construction of the Plant Expression Vector

Whole plant-based transient expression of E7*-SAPKQ was assessed in 3-week-old *Nicotiana benthamiana* plants upon agroinfiltration. Plants were grown in soil in the Bio-Safety Level-2 green-house available at ENEA, under LED lighting (650 LED Lumigrow lamp; spectroradiometric data: lux 3106.5; total PAR 138.83; Watts 0.0011) with daylight integration and dark condition (16/8 h) until use. Nutrients (Idrofill base, K Adriatica, Italy) were added to water every 3 weeks.

Tomato (*S. lycopersicum* L.) cv. Micro-Tom leaf explants were used to generate hairy root clones upon infection with recombinant *A. rhizogenes* A4. Micro-Tom plants were grown in greenhouse under hydroponic conditions and LED lighting, as described for *N. benthamiana*.

The attenuated E7GGG gene (in this work indicated as E7*) was obtained from the E7 gene of HPV16 (HPV16 genome NCBI Reference Sequence: K02718), as previously described (Massa et al., 2008). The gene encoding the leaf apoplastic saporin isoform (SAP, Genbank Acc. No. DQ105520) had been previously mutagenized to abolish toxicity (SAPKQ, IQMTAE_176_AAR_179_FRY > IQMTA**K**_176_AA**Q**_179_FRY) and to obtain the pVax-E7*-SAPKQ fusion construct by fusing the E7* sequence to the 3′ end of the SAPKQ gene (Massa et al., 2011).

**Figure 1:**
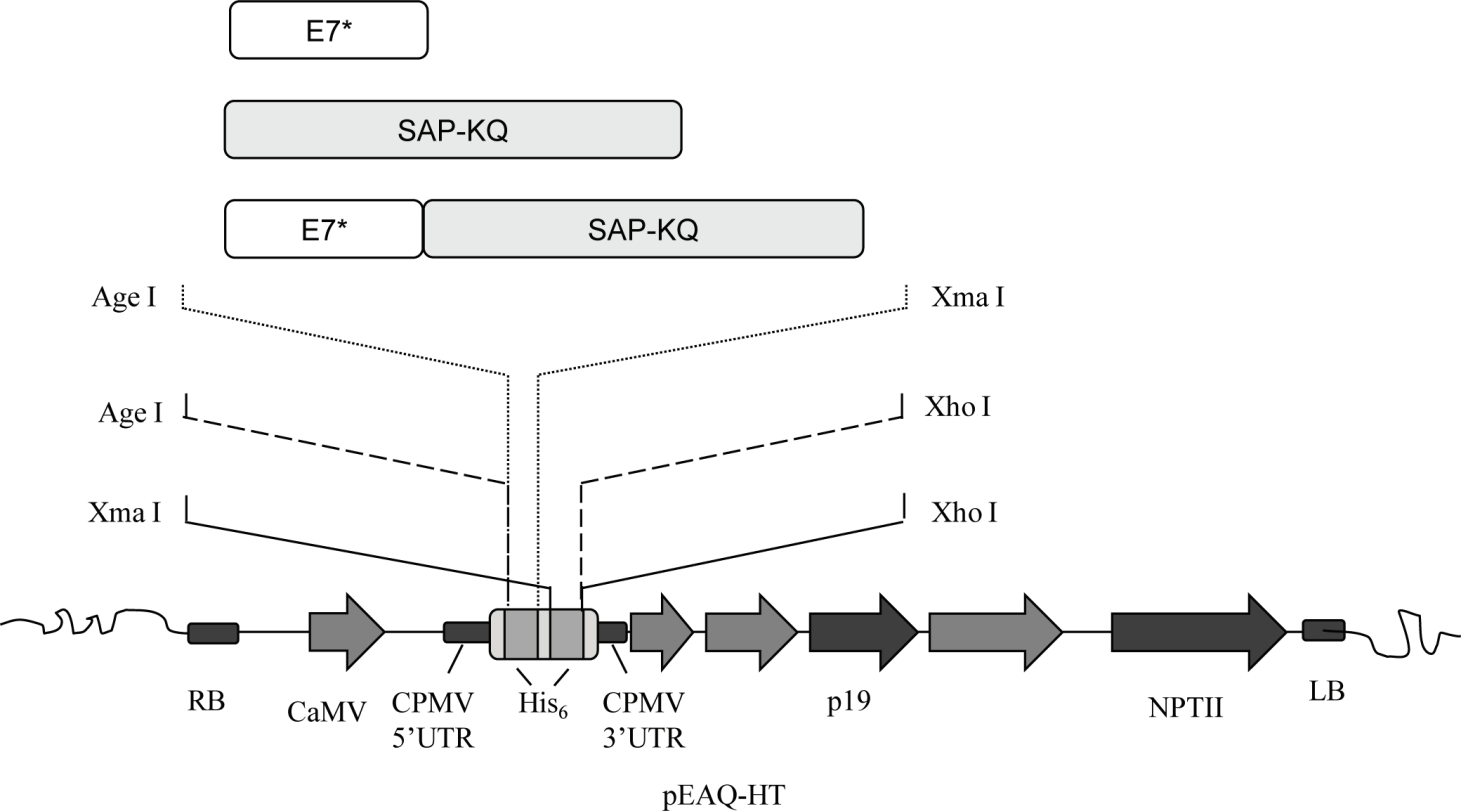
E7GGG (E7*), SAP-KQ, and E7*-SAPKQ genes were inserted in pEAQ-HT for both transient and stable plant expression. The expression cassette consisted of the cauliflower mosaic virus (CaMV) 35S promoter, the Cowpea Mosaic Virus (CPMV) 5′ and 3′ UTRs with the hyper-translatable mutation, the tomato bushy stunt virus posttranscriptional silencing inhibitor (p19), N- and C-terminal His_6_-tag for affinity purification (His_6_), and the nopaline synthase terminator sequence from *A. tumefaciens* (NPTII). Genes were cloned between the indicated sites to gain either a N-terminal, C-terminal, or no His_6_-tag after expression.

In order to obtain the plant-expression constructs, genes were PCR-amplified from the abovementioned DNA construct. Cloning into the binary Ti plasmid pEAQ-HT (PBL Technologies) (Sainsbury et al., 2009) was performed with primers designed to add either a N- or C-terminal His_6_-tag, or, on the contrary, no His_6_-tag to final products (Figure 1). Genes were inserted under the CaMV 35 promoter/Nos terminator cassette downstream of the 5′-UTR of the Cowpea Mosaic Virus (CPMV) RNA-2 harboring the U162C mutation (“hypertranslatable,” HT) and upstream of the 3′-UTR of the CPMV RNA-2. In this vector, the tomato bushy stunt virus (TBSV) p19 sequence serves as a posttranscriptional silencing suppressor.

### Agroinfiltration of Whole *N. benthamiana* Plants for Evaluation of Expression of the E7*, SAPKQ, and E7*-SAPKQ Proteins in Plant Cells

The resulting pEAQ-HT constructs were transferred into *A. tumefaciens* strain C58C1 by electroporation and, then, introduced by vacuum infiltration into *N. benthamiana* for transient expression. Briefly, 2-ml starter culture harboring the pEAQ-HT-based constructs started from a fresh colony was grown overnight in YEB containing 50 mg/l kanamycin. Then, the culture was diluted 1:500 into 500 ml of YEB, 50 mg/l kanamycin, 10 mM 2-(N-morpholino)ethanesulfonic acid (MES) pH 5.6, 2 mM MgSO_4_ and 20 μM acetosyringone, and grown overnight at 28°C, 220 rpm to O.D_600nm_ = 1.7.

Bacteria were pelleted by centrifugation at 3000 × *g* for 15 min and resuspended to a final OD_600nm_ = 2.4 with the addition of 200 μM acetosyringone in MMA medium (4.4 g/l Murashige and Skoog salts, 10 mM MES pH 5.6, 20 g/l sucrose). After 1–3 h of incubation at RT, the suspension was applied to 3-week-old *N. benthamiana* plants by vacuum infiltration cabinet (O.M.EC. Impiantistica, Grassobbio, Italy), and plants were returned to the growth module for observation. Leaves were harvested and stored at −80°C until use.

### Assessment of E7*, SAPKQ, and E7*-SAPKQ Expression in *N. benthamiana* Plants

Leaf tissues of infiltrated plants were harvested 1–7 days post infiltration. Leaf biomass samples were finely ground in liquid N_2_ with mortar and pestle, resuspended and homogenized in extraction buffer (1:3 w/v; phosphate-buffered saline “PBS”: 21 mM Na_2_HPO_4_, 2.1 mM NaH_2_PO_4_, 150 mM NaCl, pH 7.2; alternatively, GB buffer was used: 100 mM Tris-HCl pH 8.1; 10% glycerol; 400 mM saccharose; 5 mM MgCl_2_; 10 mM KCl; 10 mM 2-β-mercaptoethanol) containing a protease inhibitor cocktail (Complete™; Roche, Mannheim, Germany). Samples were incubated on ice for 30 min with gentle rocking and extracts were clarified by centrifugation at 11,000 g for 20 min. Supernatants were transferred to a fresh tube and kept on ice until use and total soluble protein (TSP) content was estimated by the Bradford assay (Bio-Rad Inc., Segrate, Italy). Pellets were resuspended in appropriate volumes of SDS-PAGE sample buffer (10% glycerol, 60 mM Tris-HCl pH 6.8, 0.025% bromophenol blue, 2% SDS, 3% 2-mercaptoethanol), constituting the insoluble fraction of leaf extracts. Samples containing 15 μg of TSP and the corresponding insoluble fraction in SDS-PAGE sample buffer were denatured at 95°C for 5 min and subsequently electrophoresed on a 12% SDS-polyacrylamide gel. Known amounts of proteins purified from *E. coli* were used as reference standards. Extract from leaves infiltrated with pEAQ-HT harboring an irrelevant gene was used as negative control. SDS Molecular Weight Standard Mixture (Sigma) was used during SDS-PAGE separation. Separated protein samples were transferred to a PVDF membrane (Millipore, Bedford, MA) by electro-transfer at 100 V with a Trans-Blot apparatus (BioRad). Filters were probed with either mouse anti-E7 or rabbit anti-SAP polyclonal antibodies (Massa et al., 2011) used at a dilution of 1:2,000 and, then, with 1:10,000 dilution of either a horseradish peroxidase-conjugated goat anti-mouse IgG antibody or a horseradish peroxidase-conjugated goat anti-rabbit IgG antibody (GE-Healthcare) for 1 h. The immune complexes were detected by developing chemiluminescence with the Immobilon Western Chemiluminescent HRP Substrate (Merck Millipore). The ImageQuant™ LAS 500 (GE Healthcare) was used for chemiluminescence signal detection and densitometric quantification of bands was performed by ImageJ software.

### Hairy Roots Generation

*S. lycopersicum* (cultivar Micro-Tom) clonal root lines were obtained from wild-type leaf explants co-cultured with *A. rhizogenes* A4 (ATCC, 43057™) harboring the constructs. Bacteria were grown in YEB medium containing 50 mg/l rifampicin and 50 mg/l kanamycin to OD600_nm_ = 0.6, at 28°C and 220 rpm. Bacteria were, then, pelleted by centrifugation at 3000 × *g* for 15 min and resuspended at OD600_nm_ = 1 in Murashige and Skoog medium (MS, Duchefa) with 30 g/l sucrose and 200 μM acetosyringone, pH 5.8.

Leaves from 3-week-old Micro-Tom plants were harvested, sterilized in 0.1% (v/v) sodium hypochlorite solution (NaClO) for 15 min, and aseptically cut into explants of 1 cm × 1 cm. Explants were subsequently inoculated by immersion in the recombinant *A. rhizogenes* suspension for 15 min, in a rotary shaker at the minimum speed, in the dark. Explants were dried onto sterilized tissue paper and transferred on their adaxial side, onto co-culture plates containing MS agar medium and 100 μM acetosyringone and incubated under dark conditions for 3 days. The co-cultured explants were, then, blotted and transferred to a hormone-free MS medium supplemented with 200 μg/l cefotaxime (Cef; Sandoz, Varese, Italy) at 25°C. Fresh growing hairy roots were obtained after 8–10 days.

Emerging roots were excised and transferred to new plates. *A. rhizogenes* was eradicated by transferring roots every 15 days onto MS agar plates using decreasing Cef concentrations (0.25, 0.125, and 0.05 g/l) until no antibiotic was added. Transformation and *A. rhizogenes* eradication were confirmed by PCR using specific oligonucleotides for the exogenous sequences and for *rol B*/*rol C* genes, and *vir C* specific primers, respectively. The selected, kanamycin-resistant hairy root clones were considered for subsequent growth and analysis. The growth rate of one representative clone for each transformation was measured as root fresh weight at different time points after liquid subculture in Erlenmeyer flasks over a 28-day culture period and this was performed on two biological replicates for each hairy root clone. Hairy root biomass harvested for subsequent analysis was carefully handled, pulverized in liquid nitrogen, and immediately stored in −80°C.

### Screening of Clonal Hairy Root Lines Expressing the Antigens by Immunoblotting Analysis

The selection of antigen-expressing hairy root clones was performed by immunoblotting. Root tissues were finely ground in liquid N_2_ with mortar and pestle and resuspended and homogenized in phosphate-buffered saline pH 7.2 (PBS, 1:3 w/v) containing a protease inhibitor cocktail (Complete™; Roche, Mannheim, Germany). Sample preparation for 12% SDS-PAGE acrylamide gels, immunoblotting, and detection of bands were performed as described for *N. benthamiana* samples. Protein molecular mass marker Color burst™ (Sigma) was used as reference for bands upon immunoblotting.

### Immunofluorescence Detection of Antigens in Hairy Roots

Recombinant His_6_-E7*-SAPKQ expressed from Micro-Tom hairy tissue samples located on glass slides was detected by immunofluorescence after softening tissues with 2% driselase in PBS for 40 min at 37°C following fixation with paraformaldehyde 4% in PBS for 1 h. Thereafter, samples were washed in PBS (pH 7.2) three times for 5 min each time with gentle shaking. Then the target was covered with 10% DMSO and 0.5% NP40 in PBS for 1 h at room temperature. Following removal of permeabilization solution, 5% BSA was applied for 1 h at RT and then, samples were dipped with primary rabbit anti-SAP polyclonal antibody diluted 1:300 and incubated at RT in wet box overnight. Then, the samples were washed three times. After a gentle drying, samples were incubated with anti-rabbit polyclonal antibody conjugated with phycoerythrin (sc-3739 goat anti-rabbit IgG-PE, Santa Cruz) diluted 1:1,000, in the dark for 1 h, washed, added with DAPI staining solution, and incubated in the dark at RT for 10 min. Subsequently, slides were washed three times in PBS. Samples were sealed with glycerol:PBS (1:1) for image collection under Nikon Eclipse TE2000-S epifluorescence microscope equipped with a Hg 100 lamp and filter sets appropriate for DAPI and Cy3 fluorescence.

### Vaccination Schedules in Mouse Model

Six- to eight-week-old female C57BL/6 mice were immunized according to two immunization protocols (Figure 6). In the first protocol (i.e., immunization protocol), mice were subjected to vaccination and, thereafter, analyzed for immune response. In the second one (i.e., therapeutic protocol), mice were subcutaneously (s.c.) injected with TC-1 tumor cells before administration of the vaccines. Immunization protocol implied a priming with the E7*-SAPKQ DNA vaccine (50 μg/mouse, intramuscularly, i.m.) into the tibia muscle, followed by electroporation according to standardized procedures (Cordeiro et al., 2015). To perform immunizations, root tissues were finely ground in liquid N_2_ with mortar and pestle, resuspended and homogenized in phosphate-buffered saline pH 7.2 (PBS, 1:3 w/v), and administered to mice. Antigen doses were quantified in the extract by immunoblotting using *E. coli*-purified fusion protein as standard, as previously described (Franconi et al., 2002). Animals were boosted after 1 week either with the same DNA- or with the E7*-SAPKQ root extract-based vaccine (1 μg/mouse, s.c. in the trunk). Alternatively, mice were primed with the E7*-SAPKQ root extract-based vaccine and boosted with the same E7*-SAPKQ root extract-based vaccine.

The therapeutic protocol implied that mice were injected with 5 × 10^4^ TC-1 tumor cells in 200-μl saline solution and, 3 days post tumor challenge, primed and boosted with the same preparations by the same time intervals used in the immunization protocol. Tumor growth was monitored by visual inspection and palpation two times a week. Animals were scored as tumor bearing when tumors reached a size of approximately 1–2 mm in diameter. For ethical reasons, the experiment was ended and all animals euthanized when tumor growth reached 3 cm^3^ in the control animals. Finally, all tumors were carefully removed from euthanized animals and tumor weight recorded.

### IFN-Gamma Enzyme-Linked Immuno-Spot Assay

HPV16 E7-specific T-cell precursors were detected by enzyme-linked immunospot assay (ELISPOT) 1 week after the boost, according to previous reported protocols (Massa et al., 2007). Briefly, single-cell suspension of splenocytes (1 × 10^6^ cells per well) from each group of vaccinated mice was added to microtiter wells coated with anti-mouse IFN-γ antibody (5 μg/ml, BD Biosciences PharMingen, San Diego, CA), along with interleukin-2 (50 units/ml, Sigma-Aldrich Italia, Milan, Italy). Triplicate samples were incubated at 37°C for 48 h with the E7-specific H-2Db (10 μg/ml) cytotoxic T-lymphocyte (CTL) MHC class I epitope (amino acids 49–57, RAHYNIVTF). After peptide incubation, a biotinylated anti-mouse IFN-γ antibody (2 μg/ml) was added for 4 h at room temperature. Cell spots were detected by streptavidin-HRP incubation for 1 h at room temperature and staining with filtered 3-amino-9-ethylcarbazole substrate (BD Biosciences PharMingen, San Diego, CA), for 5 min. Spots were counted using a dissecting microscope.

### Statistical Analysis

Comparisons between individual data points were analyzed by two-tailed Student’s *t*-test using the GraphPad Prism 8 software.^1^ Data were expressed as means ± standard deviations (SD) or ± standard error of mean (SEM). A *p* < 0.05 was considered statistically significant.

## Results

### E7*-SAPKQ Protein Expression in *N. benthamiana* Plants

To express a recombinant therapeutic HPV vaccine in plant-based systems, the E7*-SAPKQ fusion protein was cloned into the CaMV 35 promoter/Nos terminator cassette of the pEAQ-HT plant expression vector, between appropriate restriction sites, in order to add an affinity purification His_6_-tag either at the N- or C-terminus of the final product synthesized by plant cells. Also the single E7* and SAPKQ sequences were cloned in the same manner in order to verify the accumulation behavior of the single components of the fusion protein E7*-SAPKQ (Figure 1).

Constructs were introduced into plant cells either by agroinfiltration mediated by *A. tumefaciens* C58C1 in *N. benthamiana* plants for transient expression, or by transformation mediated by *A. rhizogenes* A4 to obtain stably expressing hairy root cultures from Micro-Tom leaf explants.

**Figure 2:**
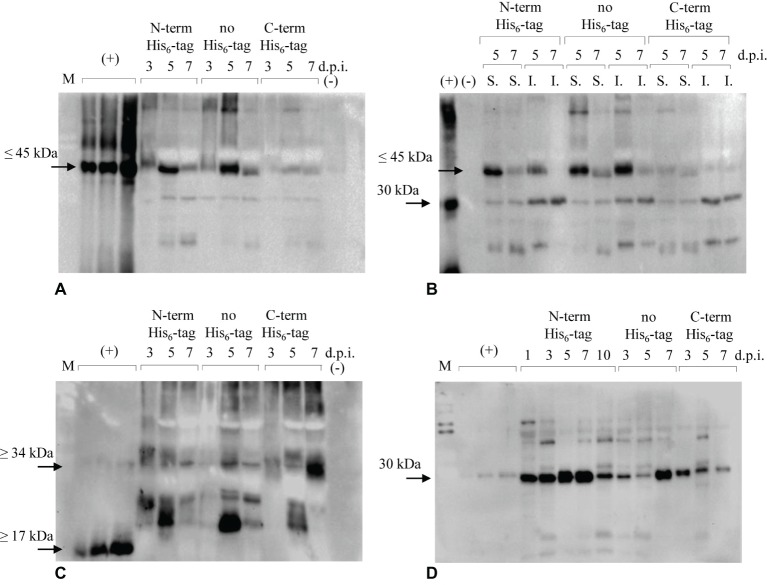
Immunoblotting analysis of accumulation of recombinant E7*-SAPKQ, E7*, and SAPKQ. **(A)** Accumulation of recombinant E7*-SAPKQ in total extracts of *N. benthamiana* infiltrated leaves (anti-SAP polyclonal antibody). (+), His_6_-E7*-SAPKQ from *E. coli* (25, 50, and 100 ng); (−), extract from leaves infiltrated with pEAQ-HT harboring an irrelevant gene; d.p.i., days post infiltration. **(B)** Soluble (S.) and insoluble (I.) fractions from leaves infiltrated with *A. tumefaciens* harboring His_6_-E7*-SAPKQ and E7*-SAPKQ. (+), His_6_-SAPKQ from *E. coli* (50 ng). **(C)** Accumulation of recombinant E7* in total extracts of *N. benthamiana* infiltrated leaves. (+), His_6_-E7* purified from *E. coli* (25, 50, and 100 ng). **(D)** Accumulation of recombinant SAPKQ in total extracts of *N. benthamiana* infiltrated leaves. (+), His_6_-SAPKQ purified from *E. coli* (1, 2.5, and 5 ng). M, SDS Molecular Weight Standard Mixture (Sigma) used during SDS-PAGE separation.

The engineered proteins were firstly introduced in *N. benthamiana* plants by transient methodology. Protein extracts from leaves were analyzed by immunoblotting over a 7-day period (from day 1 to day 7 post infiltration) to assess the accumulation of the recombinant antigens. Extractions, initially performed using PBS-based buffer, turned out to be insufficient to satisfactorily detect expression of the recombinant proteins. Then, a stronger extraction using GB buffer was used, revealing that both E7*-SAPKQ and the single antigens E7* and SAPKQ were expressed preferentially as N-terminal His_6_-tagged and non-tagged form in extracts of infiltrated leaves (Figure 2).

Among all the constructs, mainly His_6_-E7*-SAPKQ and E7*-SAPKQ were produced upon agroinfiltration (Figure 2A), and accumulated as soluble proteins in leaf biomass (Figure 2B). The theoretical weight of plant-expressed His_6_-E7*-SAPKQ and E7*-SAPKQ was confirmed by the separation of the *E. coli* standard His_6_-E7*-SAPKQ (Massa et al., 2011), showing a most distinct, specific band of the apparent molecular weight ≤ 45 kDa, that indicated the accumulation of the monomeric form of the fusion protein (Figures 2A,B). Additional faint bands at lower molecular weight are present as probable degradation by-products. Both His_6_-E7*-SAPKQ and E7*-SAPKQ accumulation show a peak 5 d.p.i. with a maximum expression level estimated of 3.20 and 3.90 μg/g fresh weight corresponding to 0.15 and 0.17% TSP, respectively. E7*-SAPKQ-His_6_ was calculated as 0.40 μg/g fresh weight.

E7* is produced upon agroinfiltration, given that a specific band is recognized by the anti-E7 antibody in plant extracts especially for the non-tagged protein 5 d.p.i. (Figure 2C). The band separates at a higher molecular weight than the *E. coli* reference standard (recombinant His_6_-E7*, about 17 kDa) (Massa et al., 2008). E7*-His_6_ seemed to accumulate at low levels 5 d.p.i., and as a dimer (about 34 kDa) 7 d.p.i. The maximum concentration calculated for His_6_-E7* and for the non-tagged E7* was 9.4 μg/g fresh weight (0.41% TSP), and 14.6 μg/g fresh weight (0.64% TSP), respectively. The dimeric E7*-His_6_ was estimated to be 8.2 μg/g fresh weight (0.36% TSP).

His_6_-SAPKQ accumulated with a peak 7 d.p.i., accounting for at least 14.52 μg/g fresh weight (0.64% TSP). Non-tagged product was found to accumulate 10.27 μg/g fresh weight (0.45% TSP), mainly 7 d.p.i. Lower expression was found for SAPKQ-His_6_ that mainly accumulated 3 d.p.i. (3.20 μg/g fresh weight; 0.14% TSP). The theoretical weight of plant-expressed SAPKQ in the three forms was confirmed by the separation of the *E. coli* standard His_6_-SAPKQ, showing a specific band of the apparent molecular weight of 30 kDa (Figure 2D).

### E7*-SAPKQ Expression in Hairy Root Cultures

After confirming the possibility to express the antigens in plant cells, the N-terminal His_6_-tagged constructs were chosen to engineer clonal hairy root lines for *in vitro*, stable, plant-based bioproduction of the experimental therapeutic vaccine. Forty-three clonal hairy root lines were isolated from *S. lycopersicum* cv. Micro-Tom after leaf explants infection with recombinant *A. rhizogenes* A4 strain transformed with the expression vector pEAQ-HT/His_6_-E7*-SAPKQ for subsequent analysis. Thirty-four hairy root clones were isolated after transformation with pEAQ-HT/His_6_-E7*. Forty-five hairy root clones were isolated after transformation with pEAQ-HT/His_6_-SAPKQ.

**Figure 3:**
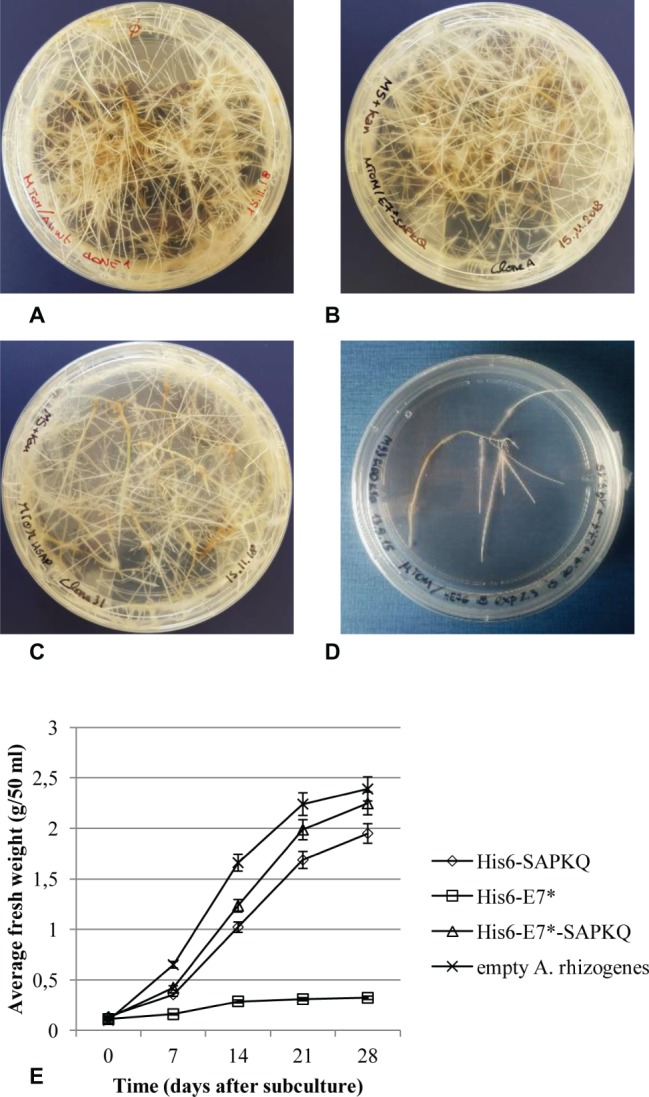
Micro-Tom (*S. lycopersicum*) clonal hairy root lines obtained after **(A)** transformation with empty *A. rhizogenes* A4, **(B)** expressing the recombinant His_6_-E7*-SAPKQ, **(C)** His_6_-SAPKQ, or **(D)** His_6_-E7*. **(E)** Growth rate of one representative clone for each transformation was measured as root fresh weight at different time points after subculture over a 28-day period. Data represent average values ± SD of triplicate assays from two biological replicates.

Hairy root clones were isolated from Micro-Tom for the different constructs and were subcultured. Recombinant hairy root clones showed slower growth rate with respect to clones obtained after transformation with non-transformed *A. rhizogenes* A4 (Figure 3A), and different growth rate depending on the construct. The best growing clones were those expressing His_6_-E7*-SAPKQ (Figure 3B), followed by those expressing His_6_-SAPKQ (Figure 3C) and by those transformed with pEAQ-HT/His_6_-E7* that had very low growth rates (Figures 3D,E).

**Figure 4:**
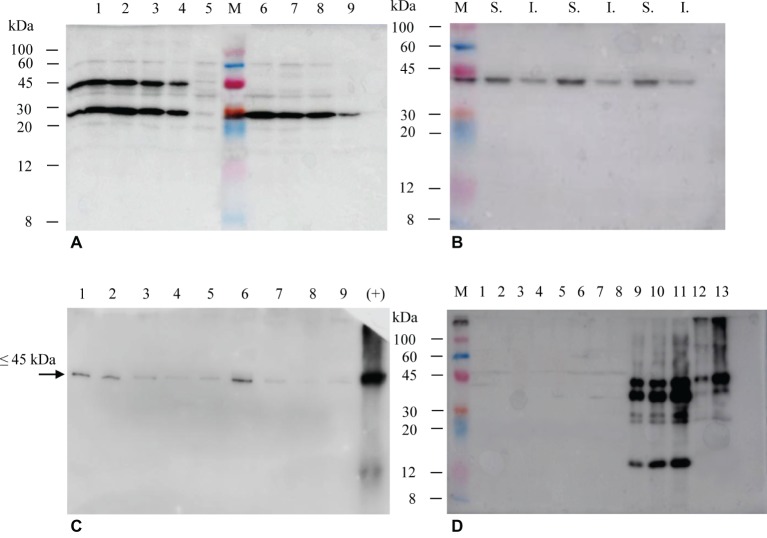
Immunoblotting of extracts (15 μg TSP) from Micro-Tom hairy root clones obtained after transformation with His_6_-E7*-SAPKQ, His_6_-SAPKQ or His_6_-E7* constructs. **(A)** Accumulation of recombinant His_6_-E7*-SAPKQ and His_6_-SAPKQ in extracts from different clonal hairy root lines (anti-SAP polyclonal antibody). Lanes 1–4, four His_6_-E7*-SAPKQ best expressing clones; lane 5, hairy root clone expressing an irrelevant gene; M, Marker Color burst™, Sigma; lanes 6–8, three His_6_-SAPKQ best expressing clones; lane 9, reference standard (His_6_-SAPKQ from *E. coli*; 25 μg); hairy root clone expressing an irrelevant gene. **(B)** Immunoblotting on soluble (S.) and insoluble (I.) fractions from extract (5 μg TSP) of three representative His_6_-E7*-SAPKQ-expressing clones (anti-E7 polyclonal antibody). **(C)** 5 μg TSP from the two best clones expressing His_6_-E7*-SAPKQ were assayed at different time points after subculture (day 7, 14, 28, 35, and 42; anti-E7 polyclonal antibody was used). Day 7, lane 1 (clone 1); day 14, lane 2 (clone 1), lane 6 (clone 2); day 28, lane 3 (clone 1), lane 7 (clone 2); day 35, lane 4 (clone 1), lane 8 (clone 2); day 42, lane 5 (clone 1), lane 9 (clone 2); lane 10, reference standard (His_6_-E7*-SAPKQ purified from *E. coli*, 100 ng; anti-E7 polyclonal antibody was used). **(D)** Assay of extracts from hairy root clones obtained after transformation with pEAQ-HT/His_6_-E7*. M, Marker Color burst™; lanes 1–8, kanamycin-resistant clones obtained after transformation and not expressing His_6_-E7*; lanes 9–11, reference standard (50, 75, 100 μg TSP from concentrated extracts of *N. benthamiana* expressing His_6_-E7*-SAPKQ); lanes 12-13, 1.5 and 5 μg TSP of one representative hairy root clone expressing His_6_-E7*-SAPKQ (anti-E7 polyclonal antibody).

The selection of hairy roots expressing the different products was performed by immunoblotting using either anti-E7* or anti-SAP polyclonal antibodies (Figure 4). In the case of His_6_-E7*-SAPKQ, 34 clones (about 79% of the isolated clones) showed the expected band at about 45 kDa indicating the presence of the full-size fusion protein. Four clones exhibited the most intense bands for the given quantity of total protein transferred (Figure 4A). Also in the case of the hairy root-produced His_6_-E7*-SAPKQ, the fusion protein accumulated mainly in the soluble fraction (Figure 4B). TSP from two out of the four best clones expressing His_6_-E7*-SAPKQ was assayed by immunoblotting analysis at different time points after subculture (day 7, 14, 28, 35, 42). As shown in Figure 4C, accumulation of His_6_-E7*-SAPKQ is stable until day 15 (i.e., along almost the whole cultivation period since clones were normally subcultured for maintenance every 21 days), to decrease thereafter. The lower weight band revealed by the anti-SAP polyclonal antibody (Figure 4A) but not by the anti-E7 polyclonal antibody (Figures 4B,C) might represent a proteolysis product containing SAPKQ, or an alternative splicing activity.

Fourteen clones resulted positive for expression of His_6_-SAPKQ (about 31% of the clones), with three clones showing the most intense bands at the expected molecular weight of about 30 kDa (Figure 4A). Among those transformed with His_6_-E7*, only eight clones survived on selective medium and none of them showed detectable expression of His_6_-E7* (Figure 4D).

All these clones were kept for subsequent subculture and measurements. His_6_-E7*-SAPKQ best clone accounted for 35.49 ± 2.69 μg/g of fresh weight (1.25% TSP) and His_6_-SAPKQ accounted for 31.06 ± 4.79 μg/g of fresh weight (0.96% TSP).

**Figure 5:**
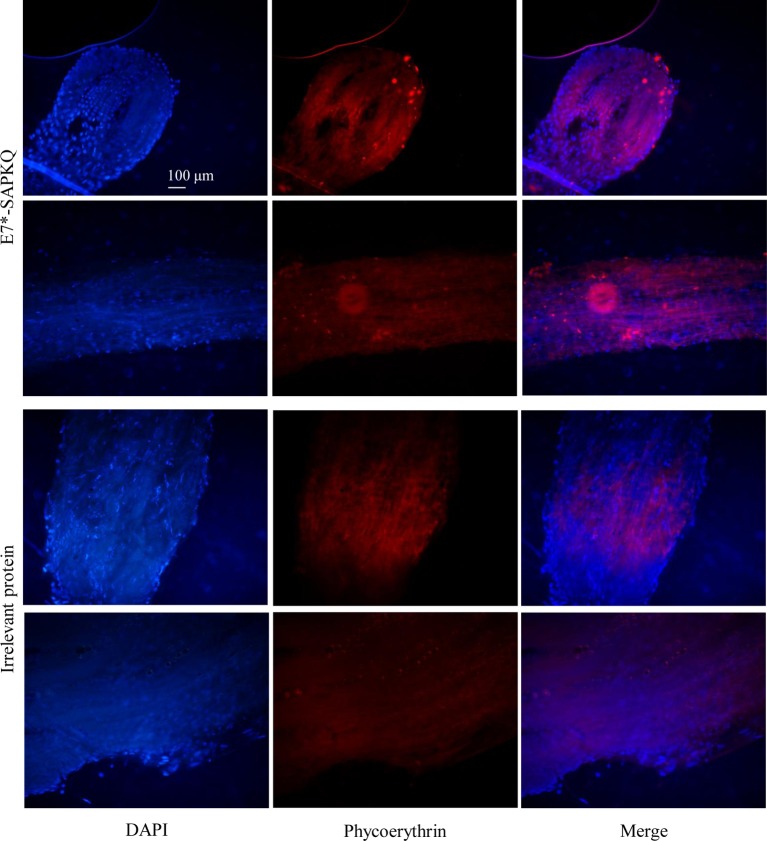
Immunofluorescence microscopic analysis showing expression of His_6_-E7*-SAPKQ in the root cap/apical meristem and along the region of elongation/maturation of a transformed hairy root clone from Micro-Tom. Original magnification 20×. Scale bar = 100 μm.

Immunofluorescence analysis performed on samples of Micro-Tom hairy root clones expressing His_6_-E7*-SAPKQ confirmed the accumulation of the recombinant protein by the detection of an intracellular specific signal. His_6_-E7*-SAPKQ (red labeling) appeared to be localized both in the root cap/apical meristem and along the region of elongation/maturation of transformed hairy roots. This showed vaccine expression both in the root tips where cell proliferation occurs and also that it was stably accumulated in mature tissues. Tissue sampling in view of preparation of extracts for administration upon preclinical studies was performed taking into account this observation. No expression of His_6_-E7*-SAPKQ in hairy roots obtained after transformation with an irrelevant gene was observed (Figure 5).

### Mouse Immunological Responses to Vaccination

**Figure 6:**
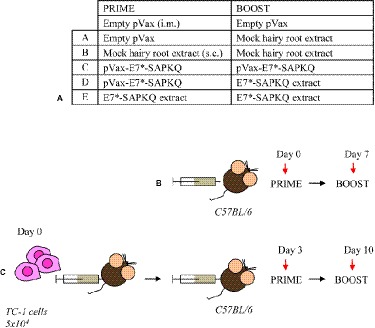
Immunization schedules. **(A)** Prime/boost regimens. A, empty pVax prime/mock hairy root extract boost; B, mock hairy root extract prime/mock hairy root extract boost; C, pVax-E7*-SAPKQ prime/pVax-E7*-SAPKQ boost; D, pVax-E7*-SAPKQ prime/E7*-SAPKQ extract boost; E, SAPKQ extract prime/SAPKQ extract boost. **(B)** Immunization of mice in the absence of tumor challenge or **(C)** in the presence of tumor challenge to evaluate immune response and tumor rejection, respectively.

**Figure 7:**
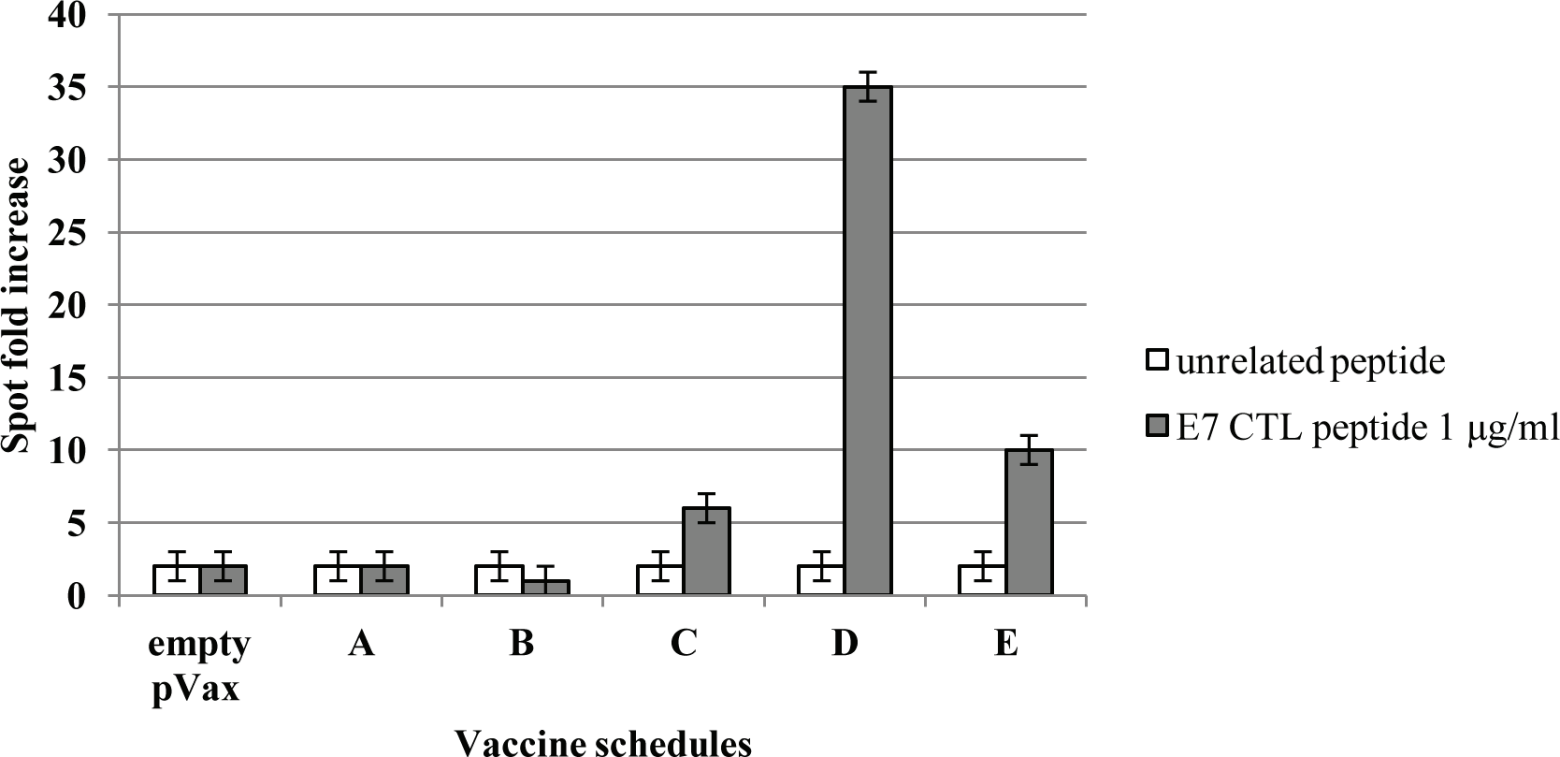
Cell-mediated immune responses in vaccinated mice. C57BL/6 mice were immunized according to schedule and procedures as in Materials and Methods. IFN-gamma production was measured in an ELISPOT assay after specific antigenic stimulation with the E7 peptide RAHYNIVTF (aa 49–57). A, empty pVax prime/mock hairy root extract boost; B, mock hairy root extract prime/mock hairy root extract boost; C, pVax-E7*-SAPKQ prime/pVax-E7*-SAPKQ boost; D, pVax-E7*-SAPKQ prime/E7*-SAPKQ extract boost; E, SAPKQ extract prime/SAPKQ extract boost. Data are presented as fold-increase responses to the E7 peptide in comparison with mice vaccinated with empty vector/irrelevant root extract, and they represent the means of all of the mice in the groups ± SD.

The immunological effects of vaccines were studied in C57BL/6 mice with the prime-boost schedule described in Figure 6. Cell-mediated immune responses were analyzed in ELISPOT assay for INF-γ secreting cells. One week after the boost, spleens were collected from immunized mice. Significantly positive scores were obtained only in the animal immunized with the root extract boost (Figure 7). In particular, heterologous prime (i.e., E7*-SAPKQ DNA)/boost (i.e., E7*-SAPKQ-containing root extract) exerted a dramatic increase in cell-mediated immune response specifically directed against E7 with respect to the other immunization combinations. The specific activity of vaccine preparation in root extracts was further confirmed by the absence of any activity of the root extracts without E7 (Figure 7).

**Figure 8:**
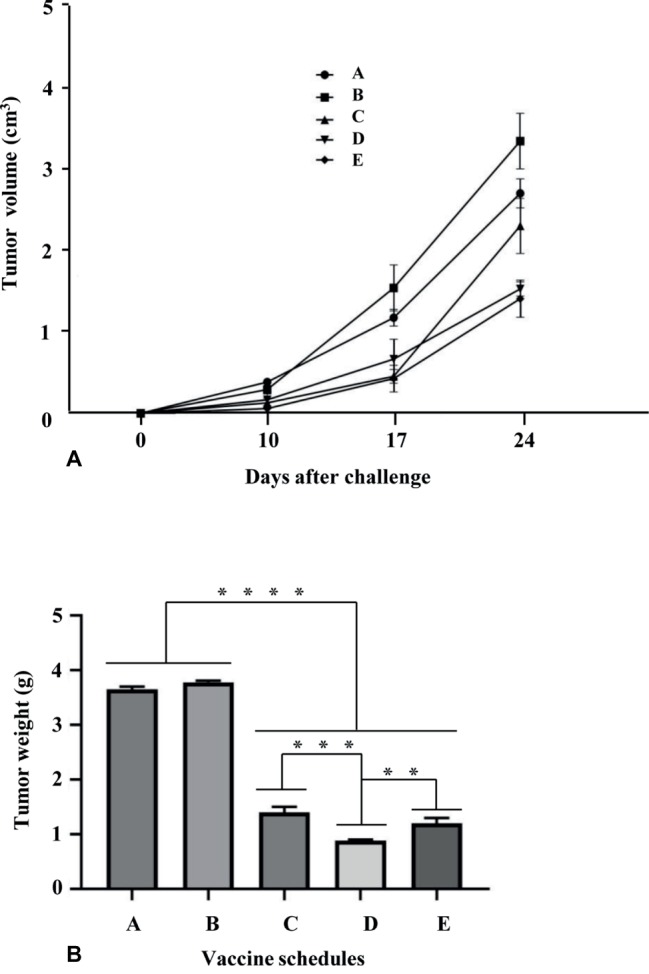
Anticancer activity His_6_-E7*-SAPKQ in TC-1 mouse model. C57BL/6 mice were challenged with 5 × 10^4^ TC-1 cells and thereafter treated with the different vaccine preparations as in Materials and Methods. Schedules of vaccination were as follows: A, empty pVax prime/mock hairy root extract boost; B, mock hairy root extract prime/mock hairy root extract boost; C, pVax-E7*-SAPKQ prime/pVax-E7*-SAPKQ boost; D, pVax-E7*-SAPKQ prime/E7*-SAPKQ extract boost; E, SAPKQ extract prime/SAPKQ extract boost. **(A)** Tumor volumes were recorded at different intervals after boost as in Materials and methods. Data represent means of five animals ± SEM. **(B)** Tumor weights were recorded when tumor controls reached >3 cm^3^ and all animals were euthanatized for ethical reasons. Data are means of five animals ± SD. ****p < 0.0001; ***p < 0.001; **p < 0.02.

The immunological responses evoked by E7*-SAPKQ-containing root extract vaccines induced us to determine if a therapeutic potential of the heterologous schedule may still be envisaged in the TC-1 preclinical model. Indeed, TC-1 cell expressing the E7 oncogene is a well-known and validated animal model for the preclinical evaluation of therapeutic candidate HPV vaccines. The homologous schedule (i.e., administration of E7*-SAPKQ DNA both as a prime and as a boost) was utilized for comparison. After injecting TC-1 cells into naïve C57BL/6 mice, prime and boost immunizations were performed on days 3 and 10, respectively (Figure 6). Tumor volume recorded 24 days after boost was mostly affected by the vaccine treatments implying the administration of the E7*-SAPKQ-containing root extracts, showing higher, statistically significant differences with respect to controls (empty pVax and mock extract, respectively) (Figure 8A). Although not statistically significant, the heterologous schedule scored a slightly higher reduction in tumor volume than the homologous administration of E7*-SAPKQ DNA both as prime and as boost.

Since tumor volume calculation *in vivo* cannot take into account all the tumor dimensions, tumors were excised from immunized mice and their weight recorded when tumor controls reached >3 cm^3^ and all animals were euthanatized for ethical reasons. Data obtained from tumor weight substantially confirmed those related to tumor volume: the heterologous E7*-SAPKQ DNA/E7*-SAPKQ-containing root extract schedule showed 4-fold reduction in tumor growth with respect to controls. In addition, the heterologous E7*-SAPKQ DNA/E7*-SAPKQ-containing root extract schedule showed statistically significant differences with respect to homologous E7*-SAPKQ DNA (both as prime and boost) or E7*-SAPKQ-containing root extracts (both as prime and boost) schedules, with *p* = 0.0009 or *p* = 0.0056, respectively (Figure 8B).

## Discussion

HPV-related cancers include cancer of the cervix, vulva, vagina, penis, or anus. HPV infection can also cause cancer in subsets of oropharyngeal tumors, including the base of the tongue and tonsils. Although HPV prevention is feasible, the effects of preventive vaccination on the incidence of HPV tumors will only be visible in the long term with a prediction of a 7.5% reduction in cases, if vaccination policies remain primarily “female-only” (Hartwig et al., 2017). In addition, current treatments are distant from the ideal ones and less effective in high-grade lesions. Expression of viral oncogenes E6, E7, and E5 leads to HPV-related malignant progression. Due to their peculiarities, HPV oncogenes represent an excellent target for cancer immunotherapy (de Freitas et al., 2017).

HPV peptide- and protein-based experimental vaccines have reached phase II trial phases, the whole protein-based having the advantage to more probably evoke efficient cell-mediated responses in patients. Nevertheless, it has been suggested that adjuvants and tools able to improve immunogenicity and endowed with less safety concerns for human health than those that are currently tested (e.g., calreticulin, lysosome-associated proteins, heat shock proteins, tetanus toxoid) are necessary to minimize autoimmune reactions, or preexisting immunity (Wolkers et al., 2002; Millar et al., 2003; Savelyeva et al., 2003). Therefore, there is still need for further improvements in terms of safety, efficacy and, possibly, of decreased costs of vaccine preparation.

Plant molecular farming (PMF) may be the approach to achieve these results. Indeed, PMF is devoted to produce active and secure cost-effective pharmaceutical proteins, and plants can be a source of immune-stimulating tools in terms of both primary and secondary metabolites. So far, production of valuable pharmaceutical proteins by PMF has been demonstrated, which can help the treatment of patients particularly in developing countries, where production and preservation costs of medicines cannot be afforded. Platforms for PMF are different and may involve the use of whole plants or plant cell/organ cultures subjected to a transient or stable expression. PMF may be intended for purification, or administration as a crude extract or whole plant tissues. All these aspects may emphasize the advantages of the plant-based systems for expression of pharmaceutical proteins. Indeed, it has been shown that genes encoding tumor-associated antigens and viral coat proteins of HPV can beexpressed in plants not only retaining their native immunological activity but also receiving adjuvant activity from plant extract itself (Franconi et al., 2002, 2006; Chabeda et al., 2018).

On the other hand, safety, efficacy, and potential immunogenicity are also features of DNA vaccines targeting HPV (Gurunathan et al., 2000; Stevenson et al., 2004; Massa et al., 2008). Literature data indicate that genetic immunotherapy is becoming a pharmacological tool and therapeutic option against cervical disease, with HPV DNA vaccines reaching encouraging results in phase II clinical trials phases (Vici et al., 2016).

We have already reported that the genetic fusion of E7* with SAPKQ modulates E7-specific humoral and cell-mediated immune responses resulting in antitumor effects against E7-expressing tumors when administered as a DNA vaccine. These findings opened the way to a new application of this plant protein, known in medicine mainly as the cytotoxic component of immuno-toxins, and gave us further impulse to the development of candidate HPV therapeutic vaccine, expanding the nature of the possible immune-enhancers of HPV E7.

This manuscript describes the use of hairy root culture expression technology for the bioproduction of a candidate therapeutic vaccine endowed with specific cell-mediated response associated to anticancer activity against HPV in a mouse model. This is the first time, to our knowledge, that such plant-based expression platform is preclinically proven to produce HPV antigens with antitumor activity.

We report expression of E7*-SAPKQ in both whole *N. benthamiana* plants by transient expression technology and hairy roots from tomato by stable transformation with the idea to develop a safe and affordable therapeutic vaccine for HPV malignancies. Our purpose was to accumulate the E7* protein in a plant-based expression system maintaining/improving its antigenicity.

Expression of the SAPKQ protein had been already tried in *E. coli* (Massa et al., 2011). Indeed, bacterial expression of SAPKQ was obtained with no bacterial growth block, demonstrating that the mutagenesis that had been introduced into the saporin leaf apoplastic isoform coding sequence of *S. officinalis* was able to highly reduce its cytotoxicity, otherwise leading to procaryotic/eucaryotic cell death (Stirpe, 2004). Nevertheless, when expression of different versions of E7*-SAPKQ fusion protein was attempted, the majority of the recombinant fusion proteins was found in the insoluble fraction of the bacterial lysates, as a possible consequence of residual toxicity of the SAPKQ proteins and of its E7GGG fused derivatives in the bacterial host (usually able to neutralize “disturbing” exogenous proteins in inclusion bodies found in the insoluble fraction of lysates upon extraction procedures). Therefore, alternative expression systems were tried, and, in particular, eukaryotic plant-based expression chassis.

The engineered proteins E7*, SAPKQ, and E7*-SAPKQ were firstly introduced into *N. benthamiana* plants by transient methodology, primarily to assess if the recombinant protein expression was generally tolerated in plant cells. The single components of the fusion were utilized to investigate if protein bioaccumulation by plant cells was in function of the type of protein itself. It is noteworthy that while E7* alone was difficult to accumulate (at least in the presence of the His_6_-tag), the different forms of SAPKQ were quite easily accumulated. This gave a clue about a possible positive influence of the SAPKQ in the accumulation of the fusion product E7*-SAPKQ. Indeed, even though at low levels, His_6_-E7*-SAPKQ was expressed in plants upon agroinfiltration in both un-tagged and His_6_-tagged forms, and turned out to be accumulated mainly as a soluble protein. This result also suggested the possibility to devise a different plant-based platform for stable transformation to possibly achieve better protein yield and purification in native conditions. However, the low and different accumulation levels of all the forms of E7* prompted us not to purify these antigens from the infiltrated leaf biomass.

We demonstrated that infection of Micro-Tom leaf explants with recombinant *A. rhizogenes* can be used for rapid establishment (approximately 6 weeks) of hairy root clones stably expressing His_6_-E7*-SAPKQ. Expression yield accounted for at least 35.5 μg/g of fresh weight. This value was tenfold higher than that obtained in whole plants upon agroinfiltration and it is comparable to or higher than those reported for other exogenous proteins accumulated in recombinant hairy roots. His_6_-E7*-SAPKQ in hairy roots yielded approximately fivefold higher than a similar fusion protein intended for immunization, the rabies glycoprotein-ricin toxin B chain (i.e., another plant toxin adapted for therapeutic use like naturally found saporin) chimera, in optimized air-lift tomato hairy root-based bioreactors (6–8 μg/g) (Singh et al., 2015). Fusion of ricin toxin B chain with F1:V pneumonic plague vaccine antigen in tobacco hairy roots yielded 140-fold less than His_6_-E7*-SAPKQ (0.25 μg/g) (Woffenden et al., 2008). De Guzman et al. (2011) reported production of the *E. coli* B-subunit heat labile toxin antigen in tomato hairy root cultures (~10 μg/g; the same protein was expressed in tobacco and petunia hairy roots ~100 μg/g).

His_6_-E7*-SAPKQ expression in tomato clonal hairy root lines was maintained along subculture intervals, and lowering of expression was observed when hairy root clones were forced to age in culture vessels. All these results demonstrate the suitability of the hairy root system to our purposes also in absence of optimization of culture conditions. It is noteworthy that a mild extraction (i.e., performed in PBS, the same solution that was subsequently used for immunizations) was sufficient to obtain the recombinant proteins, while a stronger buffer was necessary to extract the same products from leaf tissues.

No hairy root clones expressing His_6_-E7* alone were selected. This is not surprising since, in contrast to transient expression, that was demonstrated several times for E7 in plant-based expression systems (reviewed in Chabeda et al., 2018), constitutive expression could imply some toxicity affecting the onset and subsequent survival of hairy root clones upon stable transformation. Actively growing clones were, on the contrary, obtained for His_6_-SAPKQ, as for His_6_-E7*-SAPKQ. This suggests also that the SAPKQ carrier may be helpful in determining a growth-compatible bioaccumulation of the candidate vaccine in the root tissues.

It is well known that heterologous DNA prime followed by recombinant protein boost regimens can be used to enhance HPV therapeutic vaccine potency (Smyth et al., 2004; Fiander et al., 2006; Peng et al., 2016; Chabeda et al., 2018).

Indeed, in this work, the boosting strategy with the E7*-SAPKQ-containing extract was effective in strongly improving E7-specific T-cell responses and related anticancer activity after E7*-SAPKQ DNA-based priming with respect to homologous prime-boost regimens, as proven by ELISPOT assay and biological tests. The model chosen to test the efficacy of these experimental therapeutic vaccines was the well-known and widely used TC-1 model. TC-1 cells are immortalized mouse lung epithelial cells transduced with HPV E6 and E7 genes, able to express these viral oncoproteins that are continuously processed through the proteasome pathway. This processing allows their epitopes to be exposed in the context of the MHC class I complex located on the cell membrane, mimicking the mechanism leading to epitope-specific killing by CTLs upon integration of the HPV genome in an infected target cell (Feltkamp et al., 1995).

E7-specific immunological and anticancer responses were also obtained with the homologous prime-boost regimen based on E7*-SAPKQ-containing hairy root extracts, suggesting that hairy root expression system could be a useful tool in devising a therapeutic vaccine strategy against HPV. The good elicitation of specific immunity and anticancer activity was statistically significantly higher than that of the homologous prime-boost regimen based on E7*-SAPKQ DNA, already demonstrated to be effective in a previous study (Massa et al., 2011). Data from tumor weight measurement confirmed efficacy of the treatment with statistically significant differences between experimental vaccines (either homologous or heterologous prime-boost regimens with E7*-SAPKQ-based preparations) and controls.

Accumulation of E7 in hairy root tissues was demonstrated as a fusion to the LicKM carrier (Massa et al., 2009); however, in the present work, for the first time, the therapeutic potential of an E7* produced in clonal hairy root lines is determined in animal studies.

Hairy root technology offers an easy handling alternative that can be conveniently biocontained in a controlled *in vitro* environment and scaled up depending on the demand for the target protein by the use of improved bioreactors. As mentioned, important experimental pharmaceutical proteins, among which vaccine antigens and their fusions with carriers, together with enzymes, hormones, and antibodies in different formats, have been reported. The hairy root system has also the advantage to theoretically allow to regenerate transgenic plants and to retain germplasm (De Guzman et al., 2011). In particular, generation of hairy roots from *S. lycopersicum* for production of foreign proteins has the advantage that there is less hazardous accumulation of alkaloids like in the case of tobacco (Singh et al., 2015), reducing the presence of unwanted compounds in extract or purified protein administration. These characteristics are essential to acquire regulatory approval for clinical administration of hairy root-produced proteins in the future.

In addition, in the case of raw and partially purified preparations from candidate vaccine-expressing tomato hairy root clones, the presence of functional elements can be exploited. As an example, a molecular complex formulation (Tomatine) based on the natural adjuvant α-tomatine (i.e., nontoxic to humans when assumed in the amounts present in green tomatoes), was reported to stimulate antigen-specific humoral and cellular immune responses that determined protection against malaria, *Francisella tularensis* and regression of experimental tumors (Morrow et al., 2004; Zhang et al., 2006; Granell et al., 2010). Indeed, preliminary analysis of our candidate vaccine-expressing hairy roots showed the presence of α-tomatine (manuscript in preparation) which may have an additive role to that of SAPKQ in the immunological and anticancer responses observed after immunization with the E7*SAPKQ-containing hairy root extracts, possibly enhancing the overall effect. Partially purified recombinant proteins have already proven to be able to induce an immune response in animal models (reviewed in Rigano et al., 2013) and may be considered in “rationally designed” formulates containing one or more active principles (e.g., vaccine antigen, bioactive secondary metabolites, and adjuvants such as α-tomatine).

Our data show that it is possible to obtain a recombinant E7*-SAPKQ from clonal hairy root lines with immunological and anticancer activities against HPV experimental tumors, especially in combination with a DNA vaccine based on the same sequences. These results pave the way to undergo more studies on production of vaccines in plant-based expression systems and their combination with other treatment modalities for the development of effective and more specific therapeutic intervention against HPV infection and related cancer.

## Ethics Statement

Animal handling and sacrifice were performed under specific pathogen-free conditions at the Animal House of the Regina Elena National Cancer Institute. All experimental procedures were approved by the Government Committee of National Minister of Health (85/2016-PR) and were carried out in accordance with EU Directive 2010/63/EU for animal experiments.

## Author Contributions

SM is the recipient of the special Grant from MIUR (Italian Ministry of University and Research) “ENEA 5 × Mille” (Young investigator Project: New therapeutic strategies for the treatment of cancer). She planned and designed the project, assembled the plant-expression constructs, undertook *N. benthamiana* agroinfiltration and generation of hairy root clones, analyzed plant material and hairy root recombinant clones, prepared extracts for immunization, planned and wrote the paper. FP undertook large-scale preparation of DNA plasmids, immunization experiments with mice, collected and analyzed data on immune responses and biological activity of the different vaccine preparations, wrote and revised the manuscript. AV planned the immunization protocols; supervised immunization experiments; analyzed and interpreted data on immune responses and biological activity of the different vaccine preparations; planned, wrote, and revised the manuscript. RF contributed to planning of the immunization experiments and in revising the manuscript. CM (Head of the Health Technologies Division) was committed in the active search of funding and revised the manuscript.

### Conflict of Interest Statement

The authors declare that the research was conducted in the absence of any commercial or financial relationships that could be construed as a potential conflict of interest.

## Footnotes

^1^www.graphpad.com

## References

[ref1] Barros M. R. Jr. de Oliveira T. H. A. de Melo C. M. L. Venuti A. de Freitas A. C. (2018). Viral modulation of TLRs and cytokines and the related immunotherapies for HPV-associated cancers. J. Immunol. Res. 2:2912671. 10.1155/2018/2912671 PMC5954921 29854832

[ref2] Chabeda A. Yanez R. J. R. Lamprecht R. Meyers A. E. Rybicki E. P. Hitzeroth I. I. (2018). Therapeutic vaccines for high-risk HPV-associated diseases. Papillomavirus Res. 5, 46–58. 10.1016/j.pvr.2017.12.006, PMID: 29277575 PMC5887015

[ref3] Cordeiro M. N. Paolini F. Massa S. Curzio G. Illiano E. Duarte Silva A. J. (2015). Anti-tumor effects of genetic vaccines against HPV major oncogenes. Hum. Vaccines Immunother. 11, 45–52. 10.4161/hv.34303 PMC4514265 25483514

[ref4] Cordeiro M. N. De Lima R. C. P. Paolini F. Melo A. R. D. S. Campos A. P. F. Venuti A.. (2018). Current research into novel therapeutic vaccines against cervical cancer. Expert Rev. Anticancer Ther. 18, 365–376. 10.1080/14737140.2018.1445527, PMID: 29475377

[ref5] de Freitas A. C. de Oliveira T. H. A. Barros M. R. Jr. Venuti A. (2017). hrHPV E5 oncoprotein: immune evasion and related immunotherapies. J. Exp. Clin. Cancer Res. 36:71. 10.1186/s13046-017-0541-1 28545552 PMC5445378

[ref6] De Guzman G. Walmsley A. M. Webster D. E. Hamill J. D. (2011). Hairy roots cultures from different Solanaceous species have varying capacities to produce *E. coli* B-subunit heat labile toxin antigen. Biotechnol. Lett 33, 2495–2502. 10.1007/s10529-011-0710-9, PMID: 21786173

[ref7] Feltkamp M. C. Vreugdenhil G. R. Vierboom M. P. Ras E. van der Burg S. H. ter Schegget J.. (1995). Cytotoxic T lymphocytes raised against a subdominant epitope offered as a synthetic peptide eradicate human papillomavirus type 16-induced tumors. Eur. J. Immunol. 25, 2638–2642. 10.1002/eji.1830250935, PMID: 7589138

[ref8] Fiander A. N. Tristram A. J. Davidson E. J. Tomlinson A. E. Man S. Baldwin P. J.. (2006). Prime-boost vaccination strategy in women with high-grade, noncervical anogenital intraepithelial neoplasia: clinical results from a multicenter phase II trial. Int. J. Gynecol. Cancer 16, 1075–1081. 10.1136/ijgc-00009577-200605000-00020, PMID: 16803488

[ref9] Franconi R. Di Bonito P. Dibello F. Accardi L. Muller A. Cirilli A.. (2002). Plant-derived human papillomavirus 16 E7 oncoprotein induces immune response and specific tumor protection. Cancer Res. 62, 3654–3658. PMID: 12097270

[ref10] Franconi R. Massa S. Illiano E. Muller A. Cirilli A. Accardi L. (2006). Exploiting the plant secretory pathway to improve the anticancer activity of a plant-derived HPV16 E7 vaccine. Int. J. Immunopathol. Pharmacol. 19, 785–795. 16569357

[ref11] Franconi R. De Murtas O. C. Massa S. (2010). Plant-derived vaccines and other therapeutics produced in contained systems. Expert Rev. Vaccines 9, 877–892. 10.1586/erv.10.91 20673011

[ref13] Gerard C. M. Baudson N. Kraemer K. Bruck C. Garcon N. Paterson Y.. (2001). Therapeutic potential of protein and adjuvant vaccinations on tumor growth. Vaccine 19, 2583–2589. 10.1016/S0264-410X(00)00486-2, PMID: 11257396

[ref14] Granell A. Fernandez-del-Carmen A. Orzaez D. (2010). In planta production of plant-derived and non-plant-derived adjuvants. Expert Rev. Vaccines 9, 843–858. 10.1586/erv.10.80 20673009

[ref15] Guillon S. Trémouillaux-Guiller J. Pat P. K. Rideau M. Gantet P. (2006). Hairy root research: recent scenario and exciting prospects. Curr. Opin. Plant Biol. 9, 341–346. 10.1016/j.pbi.2006.03.008, PMID: 16616871

[ref16] Gurunathan S. Klinman D. M. Seder R. A. (2000). DNA vaccines: immunology, application and optimization. Annu. Rev. Immunol. 18, 927–974. 10.1146/annurev.immunol.18.1.927, PMID: 10837079

[ref17] Häkkinen S. T. Raven N. Henquet M. Laukkanen M. L. Anderlei T. Pitkänen J. P.. (2014). Molecular farming in tobacco hairy roots by triggering the secretion of a pharmaceutical antibody. Biotechnol. Bioeng. 111, 336–346. 10.1002/bit.25113, PMID: 24030771

[ref18] Hartley M. R. Lord J. M. (2004). Cytotoxic ribosome-inactivating lectins from plants. Biochim. Biophys. Acta 1701, 1–14. 10.1016/j.bbapap.2004.06.004 15450171

[ref19] Hartwig S. St Guily J. L. Dominiak-Felden G. Alemany L. de Sanjosé S. (2017). Estimation of the overall burden of cancers, precancerous lesions, and genital warts attributable to 9-valent HPV vaccine types in women and men in Europe. Infect. Agents Cancer 11, 12:19. 10.1186/s13027-017-0129-6, PMID: 28400857 PMC5387299

[ref20] Komarova T. V. Baschieri S. Donini M. Marusic C. Benvenuto E. Dorokhov Y. L. (2010). Transient expression systems for plant-derived biopharmaceuticals. Expert Rev. Vaccines 9, 859–876. 10.1586/erv.10.85 20673010

[ref21] Loh H. S. Green B. J. Yusibov V. (2017). Using transgenic plants and modified plant viruses for the development of treatments for human diseases. Curr. Opin. Virol. 26, 81–89. 10.1016/j.coviro.2017.07.019, PMID: 28800551 PMC7102806

[ref22] Lonoce C. Salem R. Marusic C. Jutras P. V. Scaloni A. Salzano A. M.. (2016). Production of a tumor-targeting antibody with a human compatible glycosylation profile in *N. benthamiana* hairy root cultures. Biotechnol. J. 11, 1209–1220. 10.1002/biot.201500628, PMID: 27313150

[ref23] Lonoce C. Marusic C. Morrocchi E. Salzano A. M. Scaloni A. Novelli F. (2018). Enhancing the secretion of a glyco-engineered anti-CD20 scFv-fc antibody in hairy root cultures. Biotechnol. J. 5:e1800081. 10.1002/biot.201800081 29975457

[ref24] Massa S. Franconi R. Brandi R. Muller A. Mett V. Yusibov V.. (2007). Anti-cancer activity of plant-produced HPV16 E7 vaccine. Vaccine 25, 3018–3021. 10.1016/j.vaccine.2007.01.018, PMID: 17280752

[ref25] Massa S. Simeone P. Muller A. Benvenuto E. Venuti A. Franconi R. (2008). Anti-tumor activity of DNA vaccines based on the human papillomavirus-16 E7 protein genetically fused to a plant virus coat protein. Hum. Gene Ther. 19, 354–364. 10.1089/hum.2007.122, PMID: 18439124

[ref26] Massa S. Skarjinskaia M. Mett V. Venuti A. Yusibov V. Franconi R (2009). “Plant platforms for producing anti-cancer therapeutic vaccines” in Poster session of ‘Plant-Based Vaccines and Antibodies Plant Expression Systems for recombinant Pharmacologics – PBVA’ Congress. (Italy: University of Verona).

[ref27] Massa S. Paolini F. Spanò L. Franconi R. Venuti A. (2011). Mutants of plant genes for developing cancer vaccines. Hum. Vaccines Immunother. 7, 147–155. 10.4161/hv.7.0.14577 21266841

[ref28] Massa S. Presenti O. Benvenuto E. (2018). “Engineering plants for the future: farming with value-added harvest” in Progress in botany. eds. Canovas F. M. Lüttge U. Matyssek R. Pretzsch H. (Switzerland: Springer International Publishing AG), 80, 65–108.

[ref29] Millar D. G. Garza K. M. Odermatt B. Elford A. R. Ono N. Li Z.. (2003). Hsp70 promotes antigen-presenting cell function and converts T-cell tolerance to autoimmunity *in vivo*. Nat. Med. 9, 1469–1476. 10.1038/nm962, PMID: 14625545

[ref30] Miralpeix B. Rischer H. Häkkinen S. T. Ritala A. Seppänen-Laakso T. Oksman-Caldentey K. M.. (2013). Metabolic engineering of plant secondary products: which way forward? Curr. Pharm. Des. 19, 5622–5639. 10.2174/1381612811319310016, PMID: 23394556

[ref31] Morrow W. J. W. Yang Y. W. Sheikh N. A. (2004). Immunobiology of the Tomatine adjuvant. Vaccine 22, 2380–2384. 10.1016/j.vaccine.2004.03.022, PMID: 15193398

[ref32] Naphatsamon U. Ohashi T. Misaki R. Fujiyama K. (2018). The production of human β-glucocerebrosidase in *Nicotiana benthamiana* root culture. Int. J. Mol. Sci. 19:E1972. 10.3390/ijms19071972, PMID: 29986415 PMC6073899

[ref33] Peng S. Qiu J. Yang A. Yang B. Jeang J. Wang J. W. (2016). Optimization of heterologous DNA-prime, protein boost regimens and site of vaccination to enhance therapeutic immunity against human papillomavirus-associated disease. Cell Biosci. 25:16. 10.1186/s13578-016-0080-z PMC4766698 26918115

[ref34] Rigano M. M. De Guzman G. M. Walmsley A. M. Frusciante L. Barone A. (2013). Production of pharmaceutical proteins in *Solanaceae* food crops. Int. J. Mol. Sci. 14, 2753–2773. 10.3390/ijms14022753, PMID: 23434646 PMC3588013

[ref35] Rischer H. Häkkinen S. T. Ritala A. Seppänen-Laakso T. Miralpeix B. Capell T.. (2013). Plant cells as pharmaceutical factories. Curr. Pharm. Des. 19, 5640–5660. 10.2174/1381612811319310017, PMID: 23394561

[ref36] Roden R. B. S. Stern P. L. (2018). Opportunities and challenges for human papillomavirus vaccination in cancer. Nat. Rev. Cancer 18, 240–254. 10.1038/nrc.2018.13, PMID: 29497146 PMC6454884

[ref37] Rybicki E. P. (2014). Plant-based vaccines against viruses. Virol. J. 11:205. 10.1186/s12985-014-0205-0 25465382 PMC4264547

[ref38] Sainsbury F. Thuenemann E. C. Lomonossoff G. P. (2009). pEAQ: versatile expression vectors for easy and quick transient expression of heterologous proteins in plants. Plant Biotechnol. J. 7, 682–693. 10.1111/j.1467-7652.2009.00434.x, PMID: 19627561

[ref39] Santos R. B. Abranches R. Fischer R. Sack M. Holland T. (2016). Putting the spotlight back on plant suspension cultures. Front. Plant Sci. 7, 60–72. 10.3389/fpls.2016.00297 27014320 PMC4786539

[ref40] Savelyeva N. Zhu D. Stevenson F. K. (2003). Engineering DNA vaccines that include plant virus coat proteins. Biotechnol. Genet. Eng. Rev. 20, 101–114. 10.1080/02648725.2003.10648039, PMID: 14997848

[ref41] Schillberg S. Raven N. Fischer R. Twyman R. M. Schiermeyer A. (2013). Molecular farming of pharmaceutical proteins using plant suspension cell and tissue cultures. Curr. Pharm. Des. 19, 5531–5542. 10.2174/1381612811319310008, PMID: 23394569

[ref42] Singh A. Srivastava S. Chouksey A. Panwar B. S. Verma P. C. Roy S.. (2015). Expression of rabies glycoprotein and ricin toxin B chain (RGP-RTB) fusion protein in tomato hairy roots: a step towards oral vaccination for rabies. Mol. Biotechnol. 57, 359–370. 10.1007/s12033-014-9829-y, PMID: 25519901

[ref43] Skarjinskaia M. Ruby K. Araujo A. Taylor K. Gopalasamy-Raju V. Musiychuk K.. (2013). Hairy roots as a vaccine production and delivery system. Adv. Biochem. Eng./Biotechnol. 134, 115–134. 10.1007/10_2013_184, PMID: 23649385

[ref44] Skeate J. G. Woodham A. W. Einstein M. H. Da Silva D. M. Kast W. M. (2016). Current therapeutic vaccination and immunotherapy strategies for HPV-related diseases. Hum. Vaccin. Immunother. 12, 1418–1429. 10.1080/21645515.2015.1136039, PMID: 26835746 PMC4964648

[ref45] Smyth L. J. Van Poelgeest M. I. Davidson E. J. Kwappenberg K. M. Burt D. Sehr P. (2004). Immunological responses in women with human papillomavirus type 16 (HPV-16)-associated anogenital intraepithelial neoplasia induced by heterologous prime-boost HPV-16 oncogene vaccination. Clin. Cancer Res. 10, 2954–2961. 15131030 10.1158/1078-0432.ccr-03-0703

[ref46] Stevenson F. K. Ottensmeier C. H. Johnson P. Zhu D. Buchan S. L. McCann K. J. (2004). DNA vaccines to attack cancer. Proc. Natl. Acad. Sci. U.S.A. 101, 14646–14652. 10.1073/pnas.0404896101 15292504 PMC521995

[ref47] Stirpe F. (2004). Ribosome inactivating proteins. Toxicon 44, 371–383. 10.1016/j.toxicon.2004.05.004, PMID: 15302521

[ref48] Streatfield S. J. Kushnir N. Yusibov V. (2015). Plant-produced candidate countermeasures against emerging and reemerging infections and bioterror agents. Plant Biotechnol. J. 13, 1136–1159. 10.1111/pbi.12475, PMID: 26387510 PMC7167919

[ref49] Trimble C. L. Morrow M. P. Kraynyak K. A. Shen X. Dallas M. Yan J.. (2015). Safety, efficacy, and immunogenicity of VGX-3100, a therapeutic synthetic DNA vaccine targeting human papillomavirus 16 and 18 E6 and E7 proteins for cervical intraepithelial neoplasia 2/3: a randomised, double-blind, placebo-controlled phase 2b trial. Lancet 386, 2078–2088. 10.1016/S0140-6736(15)00239-1, PMID: 26386540 PMC4888059

[ref50] Venuti A. Massa S. Mett V. Vedova L. D. Paolini F. Franconi R.. (2009). An E7-based therapeutic vaccine protects mice against HPV16 associated cancer. Vaccine 27, 3395–3397. 10.1016/j.vaccine.2009.01.068, PMID: 19200826

[ref51] Vici P. Mariani L. Pizzuti L. Sergi D. Di Lauro L. Vizza E.. (2014). Emerging biological treatments for uterine cervical carcinoma. J. Cancer 5, 86–97. 10.7150/jca.7963, PMID: 24494026 PMC3909763

[ref52] Vici P. Pizzuti L. Mariani L. Zampa G. Santini D. Di Lauro L.. (2016). Targeting immune response with therapeutic vaccines in premalignant lesions and cervical cancer: hope or reality from clinical studies. Expert Rev. Vaccines 15, 1327–1336. 10.1080/14760584.2016.1176533, PMID: 27063030 PMC5152541

[ref53] Woffenden B. J. Nopo L. H. Cramer C. L. Dolan M. C. Medina-Bolivar F. (2008). Expression of a ricin B:F1:V fusion protein in tobacco hairy roots: steps toward a novel pneumonic plague vaccine. Elec. J. Integr. Biosci. 3, 10–19.

[ref54] Wolkers M. C. Toebes M. Okabe M. Haanen J. B. Schumacher T. N. (2002). Optimizing the efficacy of epitope-directed DNA vaccination. J. Immunol. 168, 4998–5004. 10.4049/jimmunol.168.10.4998, PMID: 11994451

[ref55] Wongsamuth R. Doran P. M. (1997). Production of monoclonal antibodies by tobacco hairy roots. Biotechnol. Bioeng. 54, 401–415. 10.1002/(SICI)1097-0290(19970605)54:5<401::AID-BIT1>3.0.CO;2-I 18634133

[ref56] Woods R. R. Geyer B. C. Mor T. S. (2008). Hairy-root organ cultures for the production of human acetylcholinesterase. BMC Biotech. 8, 95–102. 10.1186/1472-6750-8-95, PMID: 19105816 PMC2648960

[ref57] Zarovni N. Vago R. Fabbrini M. S. (2009). Saporin suicide gene therapy. Methods Mol. Biol. 542, 261–283. 10.1007/978-1-59745-561-9_14 19565907

[ref58] Zhang X. Buehner N. A. Hutson A. M. Estes M. L. Mason H. S. (2006). Tomato is a highly effective vehicle for expression and oral immunization with Norwalk virus capsid protein. Plant Biotechnol. J. 4, 419–432. 10.1111/j.1467-7652.2006.00191.x, PMID: 17177807

